# Empathy scores amongst undergraduate medical students and its correlation to their academic performance 

**DOI:** 10.30476/JAMP.2022.93026.1512

**Published:** 2022-04

**Authors:** ARSLAAN JAVAEED, ASIFA ABDUL RASHEED, ANUM MANZOOR, QURRA-TUL AIN, PRINCE RAPHAEL D COSTA, SANNIYA KHAN GHAURI

**Affiliations:** 1 Department of Pathology, Poonch Medical College, Rawalakot, Azad Kashmir, Pakistan; 2 Department of Public Health, State University of Bangladesh, Dhaka, Pakistan; 3 Department of Emergency Medicine, Shifa International Hospital, Islamabad, Pakistan;

**Keywords:** Empathy, Physician-patient relation, Communication, Medical students

## Abstract

**Introduction:**

Empathy is one of the soft skills required for building rapport and having meaningful patient-doctor interaction. Its effect on academic performance at the undergraduate level amongst Pakistani medical students is not known. This study was done to assess the relationship between empathy and gender, and the academic performance of undergraduate medical students of Azad Kashmir.

**Methods:**

This cross-sectional study was done in Poonch Medical College, Azad Kashmir, Pakistan, from May 2018 to May 2019. The sample size for this study consisted of 200 students, determined by Krejcie and Morgan sample size determination chart. Students who agreed to participate in the study were enrolled according to convenience sampling using google forms. The Interpersonal Reactivity Index (IRI) questionnaire was utilized as the data collection tool. Data were analyzed using SPSS version 25. The ethical review board approved the study. Comparisons between males and females at the IRI questions level, IRI domains level, and total IRI were made using the Mann-Whitney U test. The Spearman Rank Correlation test was used to assess the correlation between academic performance, IRI domains, and total IRI scores. A one-way ANOVA test was done to assess the relationship between academic performance and their study years.

**Results:**

A total of one hundred and fifty-one students (males 83, females 68, response rate 75.5%) participated in the study from the third to the final year of medical college. The mean empathy scores of males and females were 90.76 ± 8.39 and 91.72 ± 9.76 (p-value = 0.552). On the empathic concern scale, female students had significantly higher empathy scores (25.44 ± 3.49) than males (23.78 ± 3.88) (p-value=0.008). No significant correlation was found between the empathy scores and academic performance, using Spearman’s correlation test (p>0.05).

**Conclusion:**

Females showed significantly higher empathy scores than males for two Interpersonal Reactivity Index items and empathic concern scale. Overall, male and female students had similar total empathy scores. The relationship between empathy and academic performance was non-significant.

## Introduction

In medical sciences, empathy indicates the cognitive understanding of a patient's medical condition, emotions, and experiences from the physician’s point of view and to converse these feelings to the patient with appropriate facial expression and body language ( 1 , 2 ). Empathy is an essential component of the patient care and doctor-patient relationship ( 3 ). The ability to show proper empathy is required to be declared successful, particularly in the clinical encounter component of medical examinations, for example, the Membership of Royal College of Physicians Examination ( 4 ). Therefore, assessing empathy levels among medical students is essential.

Previous studies have found a significant relationship between empathy and gender. Females were observed to have higher empathy than males ( 5 , 6 ). These studies were done in different countries with different demographics. Moreover, previous researchers have used the 'Jefferson scale of empathy (JSE)' scale to measure empathy. The current study used Interpersonal Reactivity Index (IRI) to measure empathy among medical students. The IRI is more elaborate as it can measure the cognitive and emotional attributes, while JSE predominantly measures cognitive characteristics ( 7 ). The current study aimed to determine the empathy score differences between male and female students in Azad Kashmir according to IRI. Empathy is one of the soft skills that helps medical students view their patients as humans and not just diseases. In professional life, empathy helps with building a rapport with the patient and leads to better interaction. The present study aimed to determine the empathy scores of medical students and the way this would guide in developing curriculum.

According to previous studies, the relationship between empathy and academic performance is inconclusive ( 8 , 9 ). It means some of the researchers could find a significant relationship between empathy and academic performance, while others could not establish any significant relationship. If clinical competence is concerned, empathy plays an important role ( 10 ). After an extensive literature review, no similar previous studies were found in Azad Kashmir, Pakistan. The current study is an attempt to fill this research gap by establishing a relationship between empathy and academic performance in Azad Kashmir.

## Methods

This is a cross-sectional study conducted from May 2018 to May 2019. The study population consisted of ungraduated medical students of Poonch Medical College, Azad Kashmir, Pakistan. Currently, 300 (third to the fifth year) students study there. The study sample size was determined using Krejcie and Morgan's sample size determination chart. This chart has been well validated and used in multiple previous pieces of research for sample size calculation ( 11 - 13 ). Krejcie and Morgan introduced an estimate for sample size calculation for a finite population by using a formula ( 11 ). It is a simple method to determine the sample size based on the size of the population. According to this chart, the minimum required sample size for a population of 300 was 92. Keeping in mind an arbitrary response rate of 50%, we selected Identification numbers from the medical college of 200 students using the random number generator in SPSS.

Study questionnaires were sent to the selected students through Google Forms. The current study questionnaire was derived from Interpersonal Reactivity Index. This questionnaire was also previously validated and used by many researchers ( 14 , 15 ). This questionnaire consists of 28 five-point Likert scale-type questions to measure empathy. These questions are equally divided into four IRI domains - The perspective-taking scale, Fantasy scale, Empathic concern scale, and Personal distress scale. Their academic performance was measured by the percentage score obtained in their last professional examinations. One-way ANOVA test was performed to assess the relationship between academic performance and their study years.

After checking for completeness and correctness of the data, the data were entered in SPSS version 25 (IBM SPSS Statistics for Windows, Version 25.0. Armonk, NY: IBM Corp) for analysis. The scores for four IRI score domains (Perspective-taking scale, Fantasy scale, Empathic concern scale, and Personal distress scale) and total IRI score were calculated. The distribution of scores for all domains of IRI and total IRI score was non-normal according to the Shapiro-Wilk test. Therefore, comparisons between males and females at the IRI questions level, IRI domains level, and total IRI were made using the Mann-Whitney U test. The Spearman Rank Correlation test assessed the correlation between academic performance, IRI domains, and total IRI scores.

### Ethical Consideration

This study was approved by the institutional committee for ethical review at Poonch Medical College, Pakistan.

## Results

A total of 200 students were selected and consent forms were sent. Out of 200 students, 151 responded with a response rate of 75.5%. There were eighty-three male students (55%) and sixty-eight female students (45%), from third to the fifth year. 

The 28-item questionnaire is shown in Table 1, which shows how the students answered those questions with mean scores and standard deviation. The questions specific to each domain are indicated in Table 2. 

**Table 1:** The mean interpersonal reactivity index questions scores

Item No.	Questions	Mean	SD
1	I daydream and fantasize, with some regularity, about things might happen to me.	3.24	1.11
2	I often have tender concerned feelings for people less fortunate than me.	3.43	1.07
3	I sometimes find it difficult to see things from the other guy’s point of view.	3.17	1.07
4	Sometimes I don’t feel sorry for other people when they are having problems.	2.58	1.14
5	I really get involved with the feelings of the characters in a novel.	3.28	1.19
6	In emergency situations, I feel apprehensive and ill-at-ease.	3.28	1.96
7	I am usually objective when I watch a movie or play and I don’t get completely caught up in it.	3.28	1.03
8	I try to look at everybody’s side of a disagreement before decide.	3.40	1.08
9	When I see someone being taken advantage of, I feel kind of protective towards them.	3.43	1.08
10	I sometimes feel helpless when I am in the middle of a very emotional situation.	3.45	1.15
11	I sometimes try to understand my friends better by imagining how things look from their perspective.	3.69	0.93
12	Becoming extremely involved in a good book or movie is somewhat rare for me.	2.97	1.19
13	When I see someone get hurt, I tend to remain clam.	2.76	1.20
14	Other people’s misfortunes do not usually disturb me a great deal.	2.63	1.17
15	If I am sure, I am right about something, I do not waste much time listening to other people’s arguments.	3.52	1.11
16	After seeing a play or movie, I have felt as though, I were one of the characters.	2.90	1.24
17	Being in a tense emotional situation scares me.	3.27	1.18
18	When I see someone being treated unfairly, I sometimes do not feel very much pity for them.	2.43	1.19
19	I am usually pretty effective in dealing with emergencies.	3.60	2.62
20	I am quite touched by things, I see happen.	3.58	0.96
21	I believe that there are two sides to every question and try to look at them both.	3.76	1.00
22	I would describe myself as a pretty soft- hearted person.	3.74	1.03
23	When I watch a good movie, I can easily put myself in place of leading character.	3.15	1.13
24	I tend to lose control during emergencies.	2.78	1.01
25	When I am upset at someone, I usually put myself in his shoes for a while.	3.06	1.16
26	When I am reading an interesting story or novel, I imagine how I would feel if the events in the story were happening to me.	3.31	1.03
27	When I see someone who badly needs help in an emergency, I go to pieces.	3.35	0.97
28	Before criticizing somebody, I try to imagine how I would feel if I were in their place.	3.77	1.01

**Table 2:** Distribution of the IRI question items into four domains

Items	IRI domains
3*,8,11,15*,21,25,28	Perspective – taking scale
1,5,7*,12*,16,23,26	Fantasy scale
2,4,*,9,14*,18*,20,22	Empathic concern scale
6, 10, 13*,17,19*,24,27	Personal distress scale

The comparison of scores between male and female students is displayed in Table 3. A significant difference between the genders in terms of mean IRI item scores existed for two items: item number two, *“I often have tender concerned feelings for people less fortunate than me”* (p-value = 0.023), and item number nine *“When I see someone being taken advantage of, I feel kind of protective towards them”* (p-value = 0.038). On both occasions, females scored higher than males.

**Table 3:** Relationship between IRI question scores and gender

Item No.	Male		Female		p
	Mean	SD	Mean	SD	
1	3.22	1.06	3.26	1.18	0.823
2	3.25	1.12	3.65	0.96	0.023
3	3.08	1.08	3.28	1.05	0.231
4	2.58	1.13	2.59	1.16	0.974
5	3.28	1.12	3.29	1.28	0.867
6	3.28	2.52	3.28	0.90	0.165
7	3.20	0.98	3.37	1.08	0.297
8	3.36	1.08	3.46	1.08	0.620
9	3.25	1.16	3.65	0.94	0.038
10	3.34	1.15	3.59	1.14	0.165
11	3.61	1.00	3.78	0.84	0.396
12	3.00	1.24	2.94	1.14	0.816
13	2.76	1.23	2.76	1.17	0.931
14	2.81	1.26	2.41	1.01	0.066
15	3.54	1.09	3.50	1.15	0.915
16	2.90	1.24	2.90	1.25	0.949
17	3.28	1.15	3.26	1.22	0.980
18	2.49	1.27	2.35	1.09	0.662
19	3.81	3.39	3.34	1.06	0.392
20	3.46	1.00	3.72	0.90	0.088
21	3.80	0.96	3.72	1.06	0.719
22	3.70	1.04	3.78	1.02	0.633
23	3.28	1.04	2.99	1.23	0.121
24	2.87	1.04	2.68	0.97	0.250
25	3.14	1.16	2.96	1.15	0.317
26	3.33	1.01	3.29	1.07	0.949
27	3.39	1.00	3.31	0.95	0.794
28	3.83	1.02	3.71	0.99	0.369

IRI: Interpersonal Reactivity Index

A comparison was made between the genders to see any difference in each domain (Table 4). Among the IRI domains, only the 'Emphatic concern scale' score of females was significantly higher than that of males (p-value= 0.008). The total IRI score was similar between males and females. The correlation between academic performance and the IRI scale or any IRI domain was not statistically significant (all p-values > .050), as seen in Table 5.

**Table 4:** Relationship among IRI domains, total IR score, and gender

IRI domains	Male		Female		p
	Mean	SD	Mean	SD	
Perspective-taking scale	24.20	3.33	23.84	3.61	0.537
Fantasy scale	21.80	3.93	21.43	4.86	0.583
Empathic concern scale	23.78	3.88	25.44	3.49	0.008
Personal distress scale	21.96	4.04	22.01	3.49	0.733
Total IRI score	90.76	8.39	91.72	9.76	0.552

IRI: Interpersonal Reactivity Index

**Table 5:** Correlation of IRI score domains, total IRI score, and academic performance

		Marks secured in last professional examination	Total Score	Perspective Taking Scale	Fantasy Scale	Empathic Concern Scale	Personal Distress Scale
Marks secured in final professional examination	r	1.000	-0.008	-0.006	-0.035	-0.040	-0.032
	p		0.921	0.937	0.673	0.630	0.693
Total Score	r		1.000	0.436	0.610	0.600	0.588
	p			<0.001	<0.001	<0.001	<0.001
Perspective Taking Scale	r			1.000	0.080	0.267	0.130
	p				0.326	0.001	0.113
Fantasy Scale	r				1.000	0.091	0.173
	p					0.265	0.034
Empathic Concern Scale	r					1.000	0.185
	p						0.024

IRI: Interpersonal Reactivity Index

## Discussion

This study revealed that male and female students had similar levels of empathy which was not a contributory factor towards academic performance. To the best of our knowledge, this study is the first of its kind from Azad Kashmir region of Pakistan which is a UN disputed area.

The results correlate with a recent study done in US in 2019 where apart from the IRI scores, they also looked at burnout and perceived stress among millennial medical students. Females scored higher on the empathic scale, as shown in our study as well ( 16 ). 

 A study done in India in 2017 showed a mean empathy score of 96.01 ( 17 ). A previous Pakistani study showed a mean empathy score of 42±9.60 ( 18 ). These two results are not directly comparable with those of the current research because a different questionnaire (JSE questionnaire) was used to calculate empathy in these studies. However, JSE questionnaire also has a maximum score of 140. Therefore, the percentage-wise Indian study (68.58%) is close to the current study findings. However, the previous study from Pakistan (30%) revealed a noticeably lower level of empathy than the present study. 

A study in China used the same IRI items as the current study. They showed the mean scores for perspective-taking (17.81±4.06), empathic concern (18.77±4.21), and personal distress (13.36±3.97) ( 19 ). These scores were lower than the present study findings. They showed that women had more empathy than men in all domains; similarly, the present study showed that female students had higher empathy scores than male students in the Empathetic concern domain. One finding is common in all studies; no study revealed higher empathy in males than females, which might be attributed to the difference in upbringing, cultural factors, and stereotypical roles in society. This can be considered when developing curriculum to enhance the soft skills in male students. 

No significant relationship was found between empathy and academic scores in this study. This finding is supported by many previous studies ( 8 , 9 ) and is probably due to professional examination structures. These examinations aimed to measure medical students' knowledge, but the scenario was the opposite in professional-level doctor-patient communication ( 20 ). Empathy plays a big part in the doctor-patient relationship and can result in better history taking and building rapport with the patient. 

The limitation of this study is collecting samples from only one medical college. There are other medical colleges in the Azad Kashmir region. Therefore, the study findings may not be generalizable. This study recommends further similar studies from different medical colleges, so that future researchers can get a clear comparative picture. Further studies are also required to see the correlation between empathic scores and performance of clinical encounters with patients simulated to gain better understanding. 

## Conclusion

This study revealed an overall good level of empathy among the medical students in Azad Kashmir compared to other similar studies. Female students showed higher empathy than male ones for some IRI scale items. There was no significant relationship between empathy and academic achievement. Further studies are recommended to understand how it affects patient-doctor relationship in actual clinical setting. 

## Conflict of Interest:

None Declared.
